# Using the COM-B model to identify barriers and facilitators towards adoption of a diet associated with cognitive function (MIND diet)

**DOI:** 10.1017/S1368980020001445

**Published:** 2021-05

**Authors:** Deirdre Timlin, Jacqueline M McCormack, Ellen EA Simpson

**Affiliations:** 1 School of Psychology, Ulster University, Coleraine, UK; 2 Faculty of Science, Sligo Institute of Technology, Sligo, Ireland; 3 Psychology Research Institute, Ulster University, Coleraine, UK

**Keywords:** MIND diet, COM-B model, Dementia, Adherence, Brain health

## Abstract

**Objective:**

The aim of the study was to identify components of the COM-B (capability, opportunity, motivation and behaviour) model that influences behaviour to modify dietary patterns in 40–55-year-olds living in the UK, in order to influence the risk of cognitive decline in later life.

**Design:**

This is a qualitative study using the COM-B model and theoretical domains framework (TDF) to explore beliefs to adopting the Mediterranean-DASH Intervention for Neurodegenerative delay (MIND) diet.

**Setting:**

Northern Ireland.

**Participants:**

Twenty-five participants were recruited onto the study to take part in either a focus group or an interview. Participants were men and women aged between 40 and 55 years. Participants were recruited via email, Facebook and face to face.

**Results:**

Content analysis revealed that the main perceived barriers to the adoption of the MIND diet were time, work environment, taste preference and convenience. The main perceived facilitators reported were improved health, memory, planning and organisation, and access to good quality food.

**Conclusions:**

This study provides insight into the personal, social and environmental factors that participants report as barriers and facilitators to the adoption of the MIND diet among middle-aged adults living in the UK. More barriers to healthy dietary change were found than facilitators. Future interventions that increase capability, opportunity and motivation may be beneficial. The results from this study will be used to design a behaviour change intervention using the subsequent steps from the Behaviour Change Wheel.

Maintaining healthy dietary behaviours is crucial for population health and the prevention of non-communicable disease. The most recent statistics show that there are around 850 000 people in the UK with dementia.^(1)^ The number of people with dementia is increasing because people are living longer with estimations showing that by 2025, the number of people with dementia in the UK will have increased to around 1 million^(1)^. It is estimated that by 2025, 20 % of the population will be over 65 years and, with this increased longevity, there is a need to identify potential variables such as diet to promote healthy ageing.

Many of the epidemiological studies of dietary patterns have investigated the impact of the Mediterranean diet and the DASH diet (Dietary Approaches to Stop Hypertension)^(2)^ on cognitive function^(3)^. Research found that higher adherence to the respective diets was significantly associated with less cognitive decline in midlife over a 4-month period^(4)^ and also in older adults over a 4-year period^(5)^.

The MIND diet (Mediterranean-DASH Intervention for Neurodegenerative Delay)^(6)^ is a hybrid of the Mediterranean diet^(7)^ and DASH diet. Findings from research on the Mediterranean and DASH diets suggest they may have protective effects on cardiovascular conditions that may adversely affect brain health^(6)^. Therefore, the MIND diet was designed to emphasise the dietary components and servings linked to neuroprotection and dementia prevention^(6)^. The MIND diet consists of ten healthy foods (leafy greens, other vegetables, nuts, berries, fish, poultry, olive oil, beans, whole grains and red wine) and five other foods which are to be limited (red meat, butter, cheese, pastries and sweets, and fried foods).

There has been limited research investigating the MIND diet; however, recent research with older adults found that the MIND diet can slow cognitive decline over an average of 4·7 years^(8)^. This study found that the MIND diet score was more predictive of cognitive decline than either the Mediterranean diet or DASH diet. Research found a 53 % lower risk for Alzheimer’s disease with high adherence to the MIND diet^(8)^. Furthermore, a 35 % lower risk of Alzheimer’s disease was shown for a moderate adherence to the MIND diet^(8)^, whereas no significant association with Alzheimer’s disease was shown for the Mediterranean or DASH diet^(9)^. Further support for a lower risk of cognitive decline with both moderate and high adherence to the MIND diet is shown in Adjibade *et al*. (2019). This study showed that 72 % of the large sample (6011) adhered at least moderately to the MIND diet^(10)^. Interestingly, recent research found that the MIND diet and not the Mediterranean diet protected against 12-year incidence of mild cognitive impairment and dementia in older adults^(11)^. A longitudinal study with older adults found that higher adherence to the MIND diet was associated with less cognitive decline after a 6-year follow-up^(12)^ and that greater long-term adherence to the MIND diet was associated with better verbal memory over 6 years in older adults^(13)^.

Little is known about the social, environmental and cultural perspectives of adopting the MIND diet in the UK. However, research has found that adopting a Mediterranean style diet has social, cultural and environmental barriers. Research found that participants reported British culture to be non-conducive to a Mediterranean dietary pattern^(14)^ and that factors such as time, work and convenience were barriers to consuming a Mediterranean style diet^(15,16)^. The cost of food is suggested to play a role in peoples food choices^(17)^ and that a healthy diet may be costlier than a less healthy diet^(18,19)^. Therefore, budget could be a barrier to eating a Mediterranean style diet, especially for those of low socio-economic status. However, previous research has found that while consuming a healthier diet such as increasing fruit and vegetables may be more expensive, this cost could be offset with the reduction in meat product cost^(20)^.

This study seeks to explore the perceived barriers and facilitators to adopting the MIND diet at midlife (40–55 years) in this non-Mediterranean country. This research could also add support to the dementia strategy research by exploring modifiable risk factors in the prevention of dementia, which could be applied globally.

## Theoretical framework

**Fig. 1:**
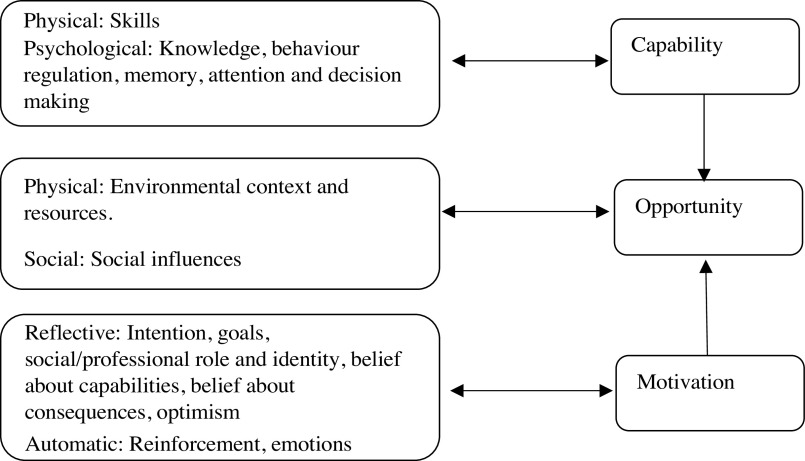
Theoretical domains framework domains and corresponding mapping onto the COM-B (capability, opportunity, motivation and behaviour) component

The behaviour change wheel is a framework for designing and evaluating interventions. At the behaviour change wheel core, there is a model of behaviour known as COM-B model, which stands for Capability (C), Opportunity (O), Motivation (M) and Behaviour (B) and posits that all three components influence behaviour, which accounts for all the factors outside the person that make the behaviour possible. The model also posits that both Capability and Opportunity influence Motivation making it the central mediator of the model; therefore, Capability and Opportunity affect behaviour both directly and indirectly. According to the COM-B model, in order to change behaviour, one or more of the COM-B components need to change, relating to either the behaviour or behaviours that support or compete with it^(21)^. In this study, the COM-B model is used to explore perceived barriers and facilitators to identify potential levers for change for adoption of the MIND diet to occur. A ‘behavioural analysis’ of the determinants of MIND diet behaviour will help define what needs to change in order for adoption of MIND diet to occur. This will be a new behaviour to many, as this diet is very new and has not been investigated in this way before. The COM-B model can be further elaborated by the Theoretical Domains Framework (TDF)^(22)^ (see Fig. 1). Although the TDF is descriptive and fails to postulate the link between domains^(23)^, it consists of fourteen domains covering the spectrum of behavioural determinants and can be mapped directly onto the COM-B components^(22)^, which specifies the relationship between domains in regard to a person’s capability, motivation and opportunity to enact a behaviour^21^ and includes constructs aligned with other behaviour change theories such as the theory of planned behaviour^(24)^. Each domain of the TDF is further elaborated by a number of core components such as belief about capabilities which include self-efficacy, control of behaviour and confidence^(22)^. The comprehensive coverage of the TDF allows researchers to analyse the most important domains specific to their target behaviour, allowing a crucial step in predicting and ultimately changing dietary behaviour. By providing a wider range of behavioural determinants, researchers gain a deeper understanding of factors influencing behaviour which can be addressed fully in intervention design.

Several qualitative studies have used the COM-B model and TDF to explore barriers and facilitators to dietary behaviour change^(25–27)^. These studies found that the COM-B model and TDF provided a comprehensive framework for describing barriers and facilitators to reducing sugar intake in young adults^(25)^, delivery of a healthy kids check to pre-schoolers^(26)^ and to athlete nutritional adherence from the sports nutritionist perspective in 26–52-year-olds^(27)^. These studies found the COM-B and TDF useful to inform an intervention to promote behaviour. Furthermore, studies have designed dietary interventions based on the COM-B model to promote the Mediterranean diet in adults at risk of CVD^(28)^, an app to improve eating habits of adolescents and young adults^(29)^ and a text messaging service targeting healthy eating for children in a family intervention^(30)^.

This study investigates the perceived barriers and facilitators to adopting the MIND diet in midlife (40–55 years). As we are looking to promote healthy ageing, we are investigating modifiable risk factors in the prevention of cognitive decline. Research has found that a good quality diet at midlife seems to be strongly linked to better health and well-being in older life^(31)^. Previous research found that adherence to a healthy dietary pattern in midlife was positively associated with cognitive functioning^(32)^.

There is currently no study investigating adoption of the MIND diet in midlife. This study addresses this gap in the literature and highlights the perceived barriers and facilitators to adopting a diet that may promote brain health at midlife and will be used to inform an intervention design.

The aim of this study was to explore perceived capability, opportunity and motivation to adopting the MIND diet among middle-aged (40–55 years) adults. The resulting information will be used to inform the design of an intervention to promote the MIND diet in middle-aged adults in the UK.

## Methods

### Design

A mixed methods qualitative design was used to elicit beliefs surrounding Capability, Opportunity, Motivation and Behaviour (COM-B) with adopting the ‘MIND’ diet. Capability, motivation and opportunity were further elaborated into fourteen domains, using a more detailed tool to understand behaviour, the TDF. Interviews and focus groups generate different information from participants. Research shows that while focus groups generate a wider range of ideas and views than that of interviews^(33)^, one-to-one interviews capture more detail than focus groups and offer more insight into participants personal thoughts and experiences^(34)^. In accordance with the COM-B framework, collecting information to understand the target behaviour, data should be collected from different sources as the most accurate picture will be informed by multiple perspectives; therefore, both focus groups and interviews were conducted^(21)^ and lasting between 30 and 60 min each (see Table 1). The interview and focus group questions were based on guidance using the COM-B^(21)^ model and TDF^(22)^ (Table 1). The model and framework were used both in developing the interview schedule and informing the content analyses used. A topic guide was developed using the TDF^(22)^. The TDF consists of a comprehensive set of fourteen domains into which all determinants of adherence to implementation of a behaviour can be organised (see Table 1). The TDF can be mapped onto the overarching COM-B model^(21)^, which posits that three key components are necessary for any behaviour—capability, opportunity and motivation.

**Table 1:** Interview/focus group questions asked to participants in accordance with the theoretical domains framework (TDF) and COM-B model

COM-B	TDF	Question
Psychological capability	Knowledge	What is your understanding of the MIND diet?
Psychological capability	Memory, attention and decision processes	To what extent is eating a diet to promote brain health something you normally do? Prompt: Do you eat foods that promote brain health each day
Psychological capability	Behaviour regulation	To what extent do you monitor whether you are eating foods that promote brain health?
Physical capability	Skills	To what extent are you confident in cooking/eating a diet that promotes brain health?
Social opportunity	Social influences	To what extent do/would your family or friends help or hinder you eating a diet that promote brain health? Prompt: Does/would your family support you in eating a diet that promotes brain health?
Physical opportunity	Environmental context and resources	Discuss anything in your work or/and home environment that might help or hinder you eating foods that promote brain health? E.g. budget and time
Reflective motivation	Social/professional role and identity	To what extent would eating a diet that promotes brain health be accepted by your friends and family? Prompt: Do you think your family/friends influences what you eat?
Reflective motivation	Belief about capabilities	How difficult/easy would it be for you to eat a diet that promotes brain health? Prompt: What are the barriers to consuming a diet that promotes brain health? Prompt: What are the facilitators to consuming a diet that promotes brain health?
Reflective motivation	Optimism	To what extent are you confident that any barriers you may have to eating a diet that promotes brain health can be solved?
Reflective motivation	Intention	To what extent do you intend to follow the MIND diet to promote brain health?
Reflective motivation	Goals	To what extent would you like to follow the MIND diet?
Reflective motivation	Belief about consequences	What do you think will happen if you eat a diet to promote brain health? Prompt: Discuss any benefits to eating a diet that promotes brain health?
Automatic motivation	Reinforcement	To what extent are there any incentives for you to eat a diet that promotes brain health?
Automatic motivation	Emotion	Discuss how you think eating a diet to promote brain health would make you feel? Prompt: Would you feel happy

COM-B: Capability (C): Psychological or physical ability to enact behaviour; Opportunity (O): Physical and social environment that enables behaviour. Motivation (M): Reflective or automatic mechanisms that activate or inhibit behaviour; Behaviour (B); MIND, Mediterranean-DASH Intervention for Neurodegenerative delay; DASH, Dietary Approaches to Stop Hypertension.

### Participants

According to similar behaviour change theories, the ideal sample size for elicitation studies is twenty-five^(23)^. Also, similar to other qualitative studies using the COM-B and TDF^(25,26)^, twenty-five participants were recruited onto the study, to take part in either a focus group or an interview. Participants were selected for interview or focus group based on their convenience to attend, which took place either in their local community hall, library, workplace or home. Participants were both Caucasian men and women aged between 40 and 55 years. Participants were recruited via email, Facebook and face to face, which took place in a supermarket. Interested participants were emailed a participant information sheet, consent form and a ‘MIND DIET’ booklet, explaining the elements of the MIND diet. Participants approached face to face were given the booklet explaining the MIND diet and asked to contact the researcher if interested in taking part, at which time, were emailed the participant information sheet and consent form. All interested participants were asked to contact the researcher by email. Dates, times and venue were arranged for focus groups and interviews.

Inclusion criteria: Male or female aged between 40 and 55-years-old living in Northern Ireland, who have no food allergies or intolerances.

Exclusion criteria: Participants following specific diets that excluded food groups, such as veganism, vegetarian and Atkins, were excluded from the study as these diets exclude foods such as fish, poultry and wholegrains, which are specific to the MIND diet. Participants with food allergies and/or intolerances were also excluded from the study.

### Procedure and materials

Participants were contacted by email, Facebook and face to face. All participants were asked to complete a personal information form which further asked if they followed a specific diet and sign the consent form before the interview/focus group began. Before interview/focus group began, there was an in-depth discussion on the MIND diet and its components between participant and researcher to ensure participants understood what the diet entailed. Participants were informed of what foods to eat, how often to eat foods and portion sizes required. There was also discussion on dementia risk factors and prevalence in the UK. The questions tapped into the components of the COM-B and TDF, that of Capability, Opportunity, Motivation and Behaviour towards consuming a healthy diet. Interviews/focus groups were approached the same in terms of discussion and questions asked and were audio recorded using a hand-held recorder.

Participants were informed that the study was voluntary and that they were free to withdraw at any time. They were assured of confidentiality regarding any personal information they supplied to the researcher.

### Data analyses

The data was transcribed verbatim and analysed using thematic analyses^(35)^. Both researchers have extensive experience and training in thematic/content analysis employed within theory of behaviour change frameworks and to inform intervention design. Researchers attended specific workshops on the COM-B framework. L.S. is a Health Psychologist and D.T. a trainee Health Psychologist, with an array of skills and experience in qualitative research analysis and the use of behaviour change theories. Two researchers independently read through the entire data set and coded the data from each transcript and assigned initial ‘code names’. Researchers kept a reflective diary to ensure a clear overview of the material. Each code was noted as either ‘barrier’ or ‘facilitator’, depending on the context in which the code occurred. There was an initial 95 % agreement of codes, which demonstrates an acceptable level of agreement^(36)^. Discussion between researchers resolved any differences within the coding process. After agreement on codes had been made, an additional step in analysis was taken by applying summative content analysis^(37)^, which involved both researchers searching the text for occurrences of codes and frequency counts for each identified code were calculated. Using a common approach^(38,39)^, TDF domains were judged based on the frequency count of coding for each TDF domain, which had been aggregated from all the factors and behaviour-specific belief statements within that domain. TDF domains were then rank-ordered according to the frequency coding to identify which components and domains of the theoretical models were the main barriers and facilitators to the adoption of the MIND diet (see Table 2).

**Table 2:** Barriers and facilitators in rank order of mentions in relation to Mediterranean-DASH Intervention for Neurodegenerative delay (MIND) diet in 40–55-year-olds: COM-B and theoretical domains framework (TDF) domains (*n* 25)^*^

COM-B	TDF	Rank order	Frequency of mentions relating to codes	% mentions†
Facilitators				
Reflective motivation	Belief about consequences	1	28	17
Reflective motivation	Belief about capabilities	2	27	16
Physical opportunity	Environment context and Resources	3	22	13
Social opportunity	Social influences	4	21	13
Physical Capability	Skills	5	20	12
Automatic motivation	Emotion	6	15	9
	Reinforcement	7	10	6
	Intention	8	6	4
	Behaviour regulation	9	4	2
	Optimism	10	4	2
	Social/professional and identity	11	3	2
	Knowledge	12	3	2
	Memory	13	1	1
	Goals	14	0	0
	Total		164	100

COM-B	TDF	Rank order	Frequency of mentions	% Mentions
Barriers				
Physical opportunity	Environment context and resources	1	90	29
Reflective motivation	Belief about capabilities	2	46	15
Psychological capability	Knowledge	3	37	12
Psychological capability	Memory, attention, decision process	4	30	10
Psychological capability	Behaviour regulation	5	24	7
Physical capability	Physical skills	6	17	6
	Social	7	15	5
	Belief about consequences	8	12	4
	Social/professional and identity	9	12	4
	Intention	10	9	3
	Optimism	11	6	2
	Goals	12	5	2
	Emotion	13	3	1
	Reinforcement	14	1	0
	Total		307	100

COM-B: Capability (C): Psychological or physical ability to enact behaviour; Opportunity (O): Physical and social environment that enables behaviour. Motivation (M): Reflective or automatic mechanisms that activate or inhibit behaviour; Behaviour (B); MIND, Mediterranean-DASH Intervention for Neurodegenerative delay; DASH, Dietary Approaches to Stop Hypertension.

* Information above the thick black line represents the top six reported domains of the TDF and corresponding COM-B components. Eighty percentage of the data fell into the top six TDF domains.

† Mentions: Spoken word/words in relation to codes/themes/subthemes emerging from questions asked regarding MIND diet.

## Results

A total of twenty-five participants took part in the study. A total of fifteen individual interviews and two focus groups were conducted to gather the data for this study. One focus group included six participants, and the second focus group included four participants. Participants were both male (40 %) and female (60 %) aged between 40- and 55-years-old with an average age of 45 years. Forty percentage of participants were of low socio-economic status. Forty-four percentage of participants had children living at home, and fifty-six percentage of participants lived rurally compared with forty-four percentage living in an urban area (see Table 3 for participants’ characteristics).

**Table 3:** Summary characteristics of interview/focus group participants (*N* 25)

	Percentage of sample	
Characteristic	%	*n*
Age (years)		
40–44	60	15
45–49	16	4
50–55	24	6
Gender		
Male	40	10
Female	60	15
Ethnicity White Irish		
Occupation	100	25
Professional	44	11
Skilled	16	4
Unskilled	40	10
Education^*^		
Higher education	36	9
Further education	28	7
No formal qualifications	36	9
Marital status		
Married	44	11
Co-habiting	4	2
Separated	4	2
Single	32	8
Widowed	4	2
Living		
Urban	44	11
Rural	56	14
Children in household		
Yes	44	11
No	56	14

* Education: level of education obtained within a discipline or profession. Higher education = undergraduate/postgraduate degree: Further education = any study after secondary school that does not include higher education, such as higher national diploma, higher national certificate, apprentices for industry such as hairdressing, plumbing.

### Theoretical framework

The transcripts provided data from all the fourteen domains of the TDF and all the components of the COM-B model. All the perceived facilitators and barriers could be fitted into one of the TDF domains and mapped onto the COM-B model, with 65 % of all mentions reported as barriers to adopting the MIND diet, compared with 35 % of mentions reported as facilitators. The most commonly reported domains were belief about consequences, belief about capabilities and environmental context/resources, and the least commonly reported domains were goals and optimism (see Tables 4 and 5 for quotes).

**Table 4:** Key facilitators, themes and quotes (*n* 25)

COM-B	TDF	Sub-theme	Quote
Reflective motivation	Belief about consequences	1. Feel better generally 2. Improve psychological health 3. Improve memory	“I think the diet would just help you feel better generally” (male 41, low education, I: P12) “And even help your head, less stress and worry” (male 55, low education, I: P21) “Well if it helps with dementia and we are heading for that” (female 40, higher education, I:14)
Reflective motivation	Belief about capabilities	1. Planning/preparation/organisation	“Organisation and preparation the night before, so having your berries and salad ready for work” (female 48, low education, I: P20) “I buy frozen cabbage, spinach, the things that I eat and just throw them in at the end and that is that” (female, 49, higher education, FG2: P8) “Preparation is a massive thing, because if you know what you are going to be eating, you can prepare for that. And you know what you are going to have for a snack or lunch”. (female 41, higher education, FG1: P4).
Physical opportunity	Environment context	1. Accessibility fresh/frozen food 2. Bring lunch to work	“I would go to Lidl, because it is cheaper and better quality” (female 40, higher education, FG1: P3) “In my work, you need to be prepared and bring lunch with you” (female 42, higher education, FG1: P5)
Social opportunity	Social influence	1. Family support/influence	“My mum is always cutting out articles showing me research on good and bad foods for your health (male 51, low education, I: P13 “I think my family would support me if I wanted to do it yes”. (male 48, low education, I: P15).
Physical capability	Skills	1. Confident cook	“I am pretty confident cooking these foods” (female 41, higher education, FG1: P6) “Well I am a confident cook, but not always the best cook, but if I see recipe, I will have a try”. (female 43, low education, I: P22) “You can google what ingredients you have and google will give you a recipe”. (female 42, higher education, FG1: P5).
Automatic motivation	Emotion	1. Positive	“I would be positive about it, I get excited trying new things” (female 50, higher education, FG2: P9) “I feel positive about it, I do intend to follow it, but not religiously, there is no point telling a lie, I am not a robot, a walking talking machine”. (male 40, low education, I: P12)

COM-B, Capability, Opportunity, Motivation, Behaviour; TDF, Theoretical Domains Framework; FG1, focus group 1; FG2, focus group 2; I, interview; P, participant.

**Table 5:** Key barriers, themes and quotes (*n* 25)

COM-B	TDF	Sub-theme	Quote
Physical opportunity	Environmental context	1. Time 2. Food environment at work/canteen 3. Budget 4. Treats in for kids.	“For me it is time, by the time you get home from work, and maybe have done overtime, you couldn’t be bothered” (male 40, further education, FG1: P1) “There is nothing healthy in a canteen” (male 50, higher education, FG2: P10) “I am on my own here with 4 kids, so budget is definitely a factor.” (female 40, low education, I: P18) “There are always buns, biscuits in the cupboards, for visitors and kids.” (female 48, further education, I: P20)
Reflective motivation	Belief about capabilities	1. Convenience 2. Taste preference 3. Mindset	“Kids don’t want healthy stuff, so sometimes I have convenience stuff to make it easier for me” (female 40, low education, I: P17) “I think if I was going to change my diet, I would have to be in the right frame of mind” (male 51, low education, I: P13) “There is stuff there I won’t eat and that is that” (male 51, further education, FG2:P7)
Psychological capability	Knowledge	1. Lack knowledge of MIND diet and foods	“If you don’t know what is healthy for your brain, you won’t eat that way” (male 40, further education, FG1: P2) “Well probably mainly cos I didn’t know it would have any benefit on my brain”. (Female 45, low education, I: P23)
Psychological capability	Memory, attention and decision process	1. Alcohol 2. Tired 3. Holidays	“If I had a drank alcohol at the weekend, it would take Tuesday or Wednesday to get over it, and I wouldn’t want to eat this food” (female 40, higher education, FG1: P3) “Well ye know, if I have been out all day with the kids and I am tired, and I haven’t the slow cooker on, there’ll be a fast food takeaway then, and that’s the reality of it”. (female 40, higher education, I: P17) “And like holidays like Christmas, you just eat for the sake of it.” (female 41, higher education, FG1: P4)
Psychological capability	Behaviour regulation	1. Lack of monitoring of food consumption	“No, I don’t, and sure, when I go to weight watchers, I don’t even do it” (female 41, low education, I: P16) “No, but trying to be very aware of it, you know, but not recording it”. (female 40, low education, I: P14)
Physical capability	Skills	1. Lack cooking skills	“I couldn’t cook that, if you handed me all the ingredients, I would be like, what am I doing with it” (male 51, further education, FG2: P7) “No, I wouldn’t be confident, I can cook basic meals, but I am not very versatile with those foods on that diet”. (male 55, low education, I: P21).

COM-B, Capability, Opportunity, Motivation, Behaviour; TDF, Theoretical Domains Framework; FG1, focus group 1; FG2, focus group 2; I, interview; P, participant.

### Capability

According to the COM-B model, for behaviour to occur, there must be the capability to do it. Capability can be either psychological (knowledge, psychological skills or stamina) to perform the behaviour or ‘physical’ (having the physical skills, strength or stamina) to perform the behaviour.

#### Psychological capability

Psychological capability was a COM-B component identified as a barrier to participants’ adoption of the MIND diet. Twenty-nine percentage of barriers to adopting the MIND diet fell into the psychological capability component of the COM-B model. These barriers also fell into three of the fourteen TDF domains, knowledge, memory, attention and decision processes, and behavioural regulation.

#### Knowledge

All participants reported that they had never heard of the MIND diet prior to the current study.

Most participants reported that they did not know that certain foods were associated with brain health.

#### Memory, attention and decision processes

The current study defined memory, attention and decision processes as the role of memory and attention to ensure adoption of the MIND diet, and ‘life distractions’, such as alcohol and tiredness, which may limit attention control with respect to eating foods that promote brain health. Several of the participants reported that alcohol is a barrier to eating brain healthy foods.

Another ‘distraction’ reported by participants was being tired. This was mainly due to participants being at work all day or having a long day with the children and too tired to cook when they came home. One participant reported eating sugary foods because of tiredness, to keep him going throughout the day.

#### Behaviour regulation

In terms of dietary patterns, behaviour regulations are the steps taken to ensure that food intake is remembered and conducted and steps taken to break unhealthy habits. In this study, most of the participants did not monitor their food intake. However, most of the participants viewed monitoring of food, with weight management programmes.

However, several participants stated that while they did not record their food intake, they were aware of what they ate.

#### Physical capability: skills

Physical skills are defined as the level of self-efficacy in cooking/eating with MIND diet foods. Six percentage of the barriers to adoption of the MIND diet fell into the TDF skills domain and mapped onto the physical capability component of the COM-B model.

Cooking skills were reported to be a barrier to adoption of the MIND diet. Those participants who reported cooking skills as a barrier tend to be married men. However, most of the participants who reported lack of cooking skills were particular to a food in the MIND diet that they usually did not eat.

Skills were also reported to be a facilitator in this study, with 12 % of all facilitators falling into the TDF skills domain. Most participants felt confident with cooking with the MIND diet foods.

Also, many participants reported that if they did not know how to cook something, they were confident that they could follow a recipe.

### Opportunity

The COM-B model states that for behaviour to occur, there must be the opportunity for the behaviour to occur in terms of a conducive physical and social environment.

#### Physical opportunity

Barriers relating to physical opportunity was the most commonly reported barrier in this study, with 29 % of all barriers falling into this component. Physical opportunity is defined in terms of what the environment facilitates in terms of time, resources, location, physical barriers etc. The TDF domain related to this component is environmental context and resources.

#### Environmental context and resources

This domain is defined as any circumstance of a person’s situation or environment that discourages or encourages the development of skills and abilities, independence, social competence and adaptive behaviour, environmental stressors, resources, salient events and person × environmental interaction. For example, cost of foods, lack of time, does not do the shopping or cooking, accessibility of cheap fresh foods.

Several participants reported that their work environment was a barrier to eating MIND diet foods. In particular, their facilities to cook at work and the canteen at work.

Time was another major barrier for most participants, especially those who were in employment. Participants reported that having worked all day, they did not have the time to cook fresh food all the time. Also, those participants who have children reported time to be a barrier. Participants reported that getting children ready for school or after school, homework and activities, took the time away from cooking healthy meals.

Having treats in the house and in the workplace is reported to be a major barrier in eating MIND diet foods. All participants with children reported having treats in for the kids but would eat the treats themselves. Also, all those participants who were employed reported that treats at work was a barrier to eating MIND diet foods. Budget was reported to be a barrier to buying some of the MIND diet foods, such as berries and nuts, as these foods are reported as expensive. This was the view of those participants who were either not working or in low paid jobs.

Environmental context and resources domain were also reported as being a facilitator to adoption of the MIND diet. Participants reported that having access to cheap fresh/frozen foods would be a facilitator. Some participants reported that, with stores like Lidl and markets where there are cheaper foods, there is really no ‘excuse’ to not eat healthy.

Participants also reported that a lot of food can be bought frozen, such as fruit, vegetables, chicken and fish and that it is cheaper and a good way of preparing meals for the week ahead. Participants also reported that a facilitator to adopt the MIND diet under this domain was to bring lunch to work. Participants felt that, in order to consume the MIND diet foods at work, they would need to bring lunch with them, to avoid eating out or from a canteen.

#### Social opportunity

Social opportunity was reported as a key facilitator in this study, with 13 % of all facilitators falling into this component. The TDF domain related to this component is social influence.

#### Social influence

Participants reported family support/influence as a key facilitator to adoption of the MIND diet. Participants reported that they felt that family would support them if they were to adopt the diet. Participants also reported that family influence would facilitate them in consuming the MIND diet.

### Motivation

Motivation is a component of the COM-B model, and there must be strong motivation for the behaviour to occur. Motivation can be divided into ‘reflective’ or ‘automated’.

#### Reflective motivation

Reflective motivation involved self-conscious planning and evaluations. (Beliefs about what is good or bad). Participants reported reflective motivation to be a barrier to the adoption of the MIND diet, and 15 % of barriers fell into this component of the COM-B model.

#### Belief about capabilities

Acceptance of the truth/reality about or validity of an ability, talent or facility that a person can put to constructive use: self-conﬁdence, perceived competence, perceived behavioural control, and self-efficacy. The extent to which the individual believes they are able to adopt the MIND diet.

Participants reported that convenience was a barrier to adoption of the MIND diet. Those participants with children reported that their children did not like healthy food or would not eat the MIND diet foods, and rather than making two meals, they ate what the children wanted out of convenience.

Taste preference was also a key barrier to the adoption of the diet under this domain. Some participants reported not liking some of the MIND diet foods, such as leafy greens, nuts or fish. Others were not willing to try different foods or try a different way of cooking those foods. Mindset was another key barrier reported to adoption of the diet within this domain. Participants reported that to change their diet and consume the MIND diet, they would have to be in the right frame of mind. They would need to want to change their diet for a reason and be determined to do so.

There were more facilitators than barriers that fell into the motivation component of the COM-B model. Forty-two percentage of the facilitators in this study fell into the motivation component of the COM-B model. Seventeen percentage of facilitators fell into the TDF belief about consequences, 16 % of facilitators fell into belief about capabilities and 9 % of facilitators fell into TDF emotion.

#### Belief about consequences

This domain is defined as the *anticipated outcomes of not eating brain healthy foods, anticipated or experienced outcomes of eating brain healthy foods (positive or negative).*

Participants reported that if they were to consume the MIND diet, they felt that this would make them feel better generally and improve memory. Some participants also reported that with the better quality of food in the MIND diet and the reduction of fat and sugar, they felt, their psychological health would improve.

#### Belief about capabilities

It was reported that in order to facilitate participants adopting the MIND diet, they would need to be prepared, organised and planned. Participants reported leading busy lives, with work and children and while time and convenience were a barrier to consuming the diet, if they were to have the MIND diet foods in the house, organise and prepare meals in advance or at least have an idea of what to cook, this would help facilitate adoption of the MIND diet.

#### Automatic motivation

Automatic motivation was reported as a facilitator to adoption of the MIND diet, with 9 % of facilitators falling into the TDF emotion domain.

Automatic motivation involves wants and needs, desires, impulse and reflex responses.

#### Emotion

Most participants reported feeling positive when asked how they feel about the prospect of adopting the MIND diet. However, this did not necessarily coincide with their intention to do so.

## Discussion

This study sought to elicit factors influencing adoption of the MIND diet in midlife in the UK. This is the first theory-based qualitative study to explore participants’ barriers and facilitators to adopting the MIND diet. Results found that 80 % of barriers and facilitators fell into six of the TDF domains, with the main barriers reported as environmental context and resources, belief about capabilities, knowledge, memory, attention and decision-making, behaviour regulation and physical skills, and the main facilitators reported as belief about consequences, belief about capabilities, environmental context and resources, social influences, skills and emotion. Results confirmed earlier findings regarding common barriers and facilitators to adopting or adherence to dietary change, including budget^(40)^, time and taste preference^(41)^, and convenience and cooking skills^(42)^.

Participants reported having no knowledge of the MIND diet prior to the study and lacked knowledge in brain healthy foods. Lacking cooking skills was also reported as a barrier, highlighting that ‘capability’ was a key barrier to adopting the MIND diet. Previous research found that a major barrier to meeting dietary recommendations was lack of knowledge regarding dietary recommendations and health benefits^(43)^ and lack of information on healthy foods^(44)^. Previous research found that not knowing what to eat or how to eat or cook healthily was a barrier to healthy eating^(45)^. Many participants reported not eating beans and lentils, which are part of the MIND diet. This was mainly due to lack of knowledge on how to prepare beans and how to make them tasty. This finding is similar to previous research that found lack of knowledge on how to prepare pulses, a barrier to their consumption^(46,47)^. Beans may not be a common staple in the Northern Irish population and, therefore, may explain why families report similar barriers regardless of income or where they live.

Participants reported a lack of monitoring their food intake which also highlights ‘capability’ as a key barrier to adoption of the MIND diet. Research found that behaviour regulation was associated with changes in dietary outcomes^(48)^ and that self-monitoring specifically showed a positive change in diet^(49)^. Maas *et al*. found that self-monitoring reduced snack eating but not alcohol consumption. However, this finding is in line with other research that suggests self-monitoring of alcohol consumptions to be weak^(50)^ or absent^(51,52)^.

Opportunity was highlighted as a barrier and facilitator to the adoption of the MIND diet, with physical opportunity reported as the main barrier. A major theme to emerge was environmental context and resources, with ‘budget’ being a significant factor, mainly due to the expense of the healthy components of the MIND diet, such as fruit, nuts and fish. Budget was only reported as a barrier by those participants who were of low socio-economic status. These findings are in line with previous research that found food cost to play an important role in determining people’s food choice and consumption^(17)^ and that it is the healthy component of a whole dietary pattern, such as fruit and nuts of the Mediterranean diet, that is associated with higher cost^(53)^. This finding is supported in the literature in a recent meta-analysis^(18)^ that found healthy foods such as fruit, vegetables and nuts to be more expensive than processed foods, refined grains and meat. Therefore, this suggests that budget could be a main barrier to adopting a healthy dietary pattern amongst those of low socio-economic status.

However, previous research compared the actual cost for a four-member family with the cost of the same family following a Mediterranean diet and found that the monthly expenditure was slightly higher on the Mediterranean diet in the overall budget^(54)^. However, after increasing the budget for fruit and vegetables, and reduced budget for processed meat and sweets, the overall budget for both diets was similar and therefore it was concluded that lower adherence to the Mediterranean diet was not related to budget, but rather, a substantial difference in allocating budget to the different food groups, for example, less money on fruit and vegetables. Similar findings were found in other research^(20,55,56)^.

Physical opportunity was also reported to be a facilitator in this study, with environmental context and resources also emerging as a theme. Access to fresh cheap produce was reported as a barrier and facilitator in the current study. The results found that those living in rural areas to be a barrier more than those living in a city, where there may be more access to markets and bigger stores within reach. Research found that stores with more nutritious food are a longer distance away from rural areas^(57,58)^. However, those who could grow their own food or had access to farmers’ markets were a facilitator to healthy eating^(59)^. Participants who received nutrition education and access to a garden to eat fruit and vegetables reported to eat the recommended daily fruit and vegetables^(60)^.

Social influence was reported as a key facilitator in this study with social influence emerging as a theme. Participants reported that family support and influence were a factor that would help them adopt the MIND diet. This finding is consistent with the previous research that found family influence as a facilitator in nutritional knowledge and healthy habit^(61)^. Other research found that those who perceived family support were more likely to eat more fruit and vegetables, wholegrains and consume less meat and fats^(62,63)^. However, family has been found to be a barrier to healthy eating^(45)^. It was reported that women were pressurised to eat more and that they were not supported if they were trying to eat a healthy diet^(45)^. However, the sample in this study was with African American women, and they may feel pressure to eat more, as food and the context of eating their traditional food is important to their cultural identity. The women in this study reported that larger curvaceous bodies are the ideal body type for African American women and that food was a big part of their customs^(45)^.

Motivation was also highlighted as a barrier and facilitator to the adoption of the MIND diet. Belief about capabilities was a major theme to emerge as a barrier. Participants reported convenience to be a factor associated with their ability to adopt the MIND diet. Previous research also found convenience to be a barrier to healthy food choices^(41)^ and that fast food and unhealthy snacks were more convenient^(59)^.

The results from this investigation have created a ‘behavioural diagnosis’ of what needs to change from the COM-B analysis in order for dietary behaviour change to occur. The COM-B model and TDF are used as a starting point to understand behaviour in the context in which it occurs. This behavioural diagnosis has identified that all three components of the COM-B model can be targeted as potential levers of change. Linking the COM-B model to the BCW allows for a systematic approach in subsequent intervention development and evaluation^(21)^. While there has been a wide range of behavioural models developed, such as the theory of planned behaviour^(24)^, they only help to understand or predict behaviour^(64)^ and do not help to understand behaviour change^(65)^ or design interventions. The behaviour change wheel guides this transition and, in designing the intervention, the COM-B components to be targeted will be mapped onto intervention functions and policy categories suggested by Michie *et al*.^(21)^ that are expected to be effective in bringing about change, such as education, persuasion and coercion. Following the identification of intervention function and policy categories, the content of the intervention will be identified in terms of which behaviour change techniques and mode of delivery are best to promote behaviour change.

### Limitations

This study was undertaken in a small sample of men and women, although in line with other COM-B studies^(66)^ and dietary studies^(67)^. Furthermore, while we were able to include participants with different sociodemographic backgrounds, this study was conducted only with a white Irish sample. However, 98 % of the population in Northern Ireland are white, with 88 % born in Northern Ireland^(68)^; therefore, the current studies’ sample reflects the majority of the Northern Ireland population. Further research to collect data from a more ethnically diverse population is needed. Moreover, our findings may be context based and, therefore, not generalisable to the whole population. However, our study did not aim to find generalisability, rather to find a deeper understanding of the people’s attitudes in midlife towards the adoption of the MIND diet that might need addressing in future interventions. Researcher subjectivity may be a limitation to our study; however, codes and themes were identified by a second researcher which suggest that the themes drawn have credence beyond interpretation of the lead researcher. Focus groups run the risk of introducing bias^(69)^, resulting from an individual’s desire to conform to social acceptability^(70)^. However, in this study, focus group participants were acquaintances and, therefore, may reduce the risk of social acceptability. Barriers and facilitators reported in this study are ‘perceived’ and, therefore, may have limited value in predicting uptake of the MIND diet. While there was a discussion on prevalence rates of dementia in the UK with participants, their perceived risk of dementia was not addressed in this study. Nevertheless, participants felt their knowledge of dementia increased, as had their knowledge of brain healthy foods. Further research should address perceived risk of dementia and its association with intention to eat a brain healthy diet.

### Strengths

The COM-B model is an established method for understanding behaviour and used extensively in behaviour change interventions, including dietary studies^(30,71)^. To our knowledge, this study is the first study to explore barriers and facilitators to adopting the MIND diet, and the first study to use the behaviour change wheel to investigate the MIND diet. This was the first study to apply the TDF to explore peoples understanding and perceptions of a whole dietary pattern. Moreover, this study used the COM-B model as an additional step in the thematic analysis, which increased the study’s efficiency and showed that the entire framework was adequate for purpose.

## Conclusion

Findings from this study provide insight into the personal, social and environmental factors that participants report as barriers and facilitators to adoption of the MIND diet among middle-aged adults living in the UK. Using the TDF and COM-B model is a starting point for understanding behaviour in specific contexts and is able to make a ‘behavioural diagnosis’ of what needs to change, to modify behaviour. The TDF and COM-B model has allowed us to gain deep understanding and increased awareness of the current situation and has clarified which barriers and facilitators can be targeted to improve adherence to the MIND diet. The results presented above suggest that there is potential to optimise all three components of the COM-B model to increase adherence to the MIND diet, highlighting the importance of addressing these factors when designing behaviour change interventions. Furthermore, understanding barriers and facilitators to the adoption of the MIND diet may help health professionals working with individuals/communities to help prevent or reduce the risk of cognitive decline. The behaviour change wheel will be used to systematically design and develop an intervention to increase adherence to the MIND diet.
